# The Impact of PM_10_ Levels on Pedestrian Volume: Findings from Streets in Seoul, South Korea

**DOI:** 10.3390/ijerph16234833

**Published:** 2019-12-01

**Authors:** Juwon Chung, Seung-Nam Kim, Hyungkyoo Kim

**Affiliations:** 1 Department of Urban Design and Studies, Chung-Ang University, Seoul 06974, Korea; juwon8995@naver.com (J.C.); snkim@cau.ac.kr (S.-N.K.); 2 Department of Urban Design and Planning, Hongik University, Seoul 04066, Korea

**Keywords:** particulate matter, air pollution, street environment, pedestrian volume, walking activity

## Abstract

Although many studies have revealed that both air quality and walking activity are dominant contributors to public health, little is known about the relationship between them. Moreover, previous studies on this subject have given little consideration to the day-to-day atmospheric conditions and floating populations of surrounding areas even though most pedestrian count surveys are not conducted on a single day. Against this backdrop, using the 2015 Pedestrian Volume Survey data and quasi-real-time weather, air quality, and transit ridership data in Seoul, this study investigates the relationship between particulate matter (PM)_10_ and pedestrian street volumes empirically. The regression results suggest that PM_10_ concentration determines people’s intention to walk and affects the volume of street-level pedestrians. The three regression models, which adopted different spatial aggregation units of air quality, demonstrated that PM_10_ elasticity of pedestrian volume is the largest in the borough-level (the smallest spatial unit of air quality alert) model. This means that people react to the most accurate information they can access, implying that air quality information should be provided in smaller spatial units for public health. Thus, strengthening air quality warning standards of PM is an effective measure for enhancing public health.

## 1. Introduction

The environment is undoubtedly one of the key components of livability [1], and in particular, air quality directly determines citizens’ quality of life by affecting individual health and outdoor activities [2]. Since the Industrial Revolution, the use of fossil fuels and the supply of automobiles have rapidly increased, and as a result, several advanced cities like London and Los Angeles have suffered from severe air pollution during the 1940s and 1950s [3]. In the U.S., smog in Los Angeles gave rise to the enactment of the Air Pollution Control Act in 1955. 

South Korea has not avoided this problem. Although air quality has consistently improved, in 2015, the yearly average PM_2.5_ concentration in South Korea was 29 µg m^−3^, which was the second-highest among the Organization for Economic Co-operation and Development (OECD) countries [4]. The World Health Organization (WHO) stated that “air pollution is an apparent environmental risk of health, and particularly particulate matter (PM) affects human health more than any other pollutants” [5], and air pollution is a key factor that increases the possibility of asthma [6,7] and cardiovascular morbidity and mortality [8,9]. Exposure to PM_2.5_ can also trigger cardiovascular diseases (CVD)-related mortality and nonfatal events, including myocardial ischemia and myocardial infarctions (MIs), heart failure, arrhythmias, and strokes [8]. Therefore, the smog in Seoul, London, and Los Angeles has been a cause of many deaths [9], and ambient air pollution is a leading contributor to global diseases, thereby affecting regional and state economies [10,11].

Although PM affects individual health, the effect of PM on walking activities has rarely been investigated [10,12]. Considering the relationship between PM, walking activities, and health levels, studying the effects of PM on walking is thought to be more helpful in understanding the health effect mechanisms of PM than directly analyzing the relationship between PM and health. Moreover, in the field of urban design, pedestrian volume on the streets is one of the most meaningful indicators [13] because it determines not only a street’s safety and vitality but also a city’s prosperity and livability [14,15]. However, little is known about the relationship between air quality (in particular, PM) and street-level walking activities, even though both influence public health. 

Against this backdrop, by using data from the 2015 Pedestrian Volume Survey (PVS) and air quality monitoring stations (AQMSs) in Seoul, this study aims to offer empirical evidence regarding the impacts of PM on the pedestrian volume on the streets. Since 2009, when the PVS was initiated, many studies have examined the determinants of pedestrian volume [16,17]. However, they did not control for weather and atmospheric conditions (in particular, PM_10_) of the survey day, although the surveys were not conducted on a single day. In addition, they rarely considered the potential floating population of the surrounding areas. Using quasi-real-time weather, air quality, and transit ridership data in Seoul, this study tackles the shortcomings of previous papers, and assuming that people react to air quality alerts from the government and media. This study also explores the differences in the spatial unit of alerts and the grade of PM_10_: “Good” (below 30 µg m^−3^), “Normal” (30–80 µg m^−3^), “Bad” (80–150 µg m^−3^), and “Very Bad” (over 150 µg m^−3^).

## 2. Literature Review

### 2.1. The Importance of Pedestrian Volume and Measuring Methods

Pedestrian volume on the streets has been considered one of the major contributors to the success of streets and cities [13]. It determines street safety and attractiveness, neighborhood livability and vitality, the prosperity of commercial districts, and the revitalization of regional and state economies [13,14,18,19,20,21]. It also enhances the physical and mental health of individuals as well as the social cohesion of the community [13,22,23,24,25,26,27]. Accordingly, various researchers have investigated how to encourage people to walk, by analyzing the relationships between built environments and pedestrian volume on the streets [16,17].

The data collection method for measuring pedestrian volume generally falls into three main groups: Self-reported surveys, information and communication technology (ICT)-aided detection (automated counting), and trained investigator observations (fieldwork and manual counting) [28]. Most traditional approaches to estimate pedestrian volume use self-reported travel diary surveys [23,29,30,31,32]. Using relatively large random samples covering major metropolitan areas or national territories, many researchers have revealed that the 5D-variables (density, diversity, design, destination accessibility, and distance to transit) are the key drivers of walking [33,34,35,36,37]. However, with this approach, measuring the pedestrian volume of each specific street is challenging and inaccurate; thus, they generally examined the relationship between the TAZ (transportation analysis zone)-level built environment and individual-level walking behaviors. Although several studies have used exact x–y coordinates of the origins and destinations of trips, they still lack information about trip routes [35], leading to low accuracy in estimating street-level pedestrian volume. The high dependency on retrospective surveys is also a key limitation of this approach [38]. 

The second type of research uses automated detection technologies, including GPS, static/mobile sensor, and video-taping and image recognition (motion detection) techniques to measure pedestrian volume. Compared to self-reported surveys, GPS obtains the exact information of pedestrian behaviors (origin, destination, and route of travel) for certain continuous periods [39], allowing for more in-depth studies that focus on the relationships between micro-level built environments and walking activity. For example, by using GPS data, Carlson et al. [40] revealed that neighborhood walkability was positively associated with walking. Moreover, GPS can be used to measure an individual’s physical activity, such as total time spent on outdoor activities [41] or certain travel modes [40]. However, because each participant needs a GPS device and they produce a large amount of data, previous studies have only applied this technology to a small number of participants of specific target group such as housewives [42], children [41], and adolescents [39], rather than a large number of general subjects. Whereas GPS is a more accurate tool than travel diary surveys, it is still inadequate for estimating pedestrian volume on streets. Rather, this tool is better suited for finding geographical activity patterns of target groups, including the frequently used street [43] or daily activity realm [39].

Infrared and thermal sensors have also been used as an automatic pedestrian counting method [44,45,46]. However, due to the unrestricted movement of pedestrians, the accuracy of this method is still underdeveloped [47]. Particularly, the technology tends to underestimate the total number of pedestrians when they move in groups [44]. It is also quite costly. 

Similarly, the computer vision technique, which automatically recognizes and counts pedestrians from still or moving images, has been used in pedestrian volume research [48]. As an example, from self-taped videos, PlaceMeter [49] extracts the metrics of pedestrian volume and other related attributes that define street quality such as waiting time in a line, temperature, noise type, level, and number of tables and chairs available. However, it also requires a large-scale deployment of recorders; thus, it is also quite costly. In addition, video recording and facial recognition technologies may run the risk of privacy invasion.

The last approach is the investigator’s field observations. Manual counting and mapping is the most traditional and intuitive way of measuring pedestrian volume. This has been widely used by the pioneers of public life studies [14,50,51]. However, due to several shortcomings of this approach, recently, the automated techniques described above have been preferred. Yin et al. [28] summarized that significant limitations of the manual counting method are the cost, time, data accuracy, subjectivity, and availability. The weakest point of this research data is that collecting large and spatially dispersed samples is extremely difficult and costly [28]. To address this limitation, some city governments have implemented large-scale public pedestrian volume surveys covering whole areas of a city [17,52]. To develop pedestrian activity and collision models, the city of Montreal collected pedestrian and traffic volumes in 519 signalized intersections [52], although this is still not as much data as compared to previous studies. However, the Pedestrian Volume Survey in Seoul, which was conducted from 2009 to 2015, surveyed 1000–10,000 points each year, covering almost all of the major streets of the city. This survey adopted the manual count, but it resolved most of the limitations described above. The investigators were hired and trained by the Seoul city government, and although the survey was conducted by a large number of people, the common standards and protocols minimized human errors and subjectivity compared to previous studies. Schneider et al. [45] also argued that manual count methods tend to be more accurate than automated count methods if the observers are properly motivated, and their fatigue is managed. Moreover, whereas pedestrian count data cannot be acquired easily as a secondary data source [28], the government opens this data to the public through the Open Data Portal [53]. This increases the reuse of the data, allowing public verification, and improving accuracy and availability. Thus, based on the data, several studies have been conducted, and the next subsection reviews them in detail. 

### 2.2. Determinants of Pedestrian Volume on the Streets

#### 2.2.1. Built Environment and Pedestrian Volume 

Using pedestrian volume data, various studies have examined the determinants of walking activity. Rodríguez et al. [54] examined the associations between built environment characteristics and observed pedestrian counts of 338 street segments around 63 Bus Rapid Transit (BRT) stations in Bogota. Their negative binomial regression results demonstrated that sidewalk width, number of crossing aids (such as signals), and road density were key determinants of pedestrian volume. Among the control variables, the BRT ridership was positively and significantly associated with the pedestrian counts, while the weather conditions (whether it rained or not) were not significant. Ewing et al. [37] also applied negative binomial regression with pedestrian counts and streetscape measurements data in 588 blocks, New York City. They revealed that not all, but some of the “D” variables (population density, floor area ratio, and distance to rail) were associated with the walking activity. Using pedestrian counts of 302 sampled street segments from 2007 to 2010 in Buffalo, New York, Hajrasouliha and Yin [55] suggested that street connectivity measures have a significant positive impact on pedestrian volumes, together with the traditional “D” variables (job density and land use mix). Miranda-Moreno et al. [52] analyzed the impacts of physical environment variables on pedestrian volumes in 519 signalized intersections in Montreal. They revealed that the conventional density and diversity variables and transit accessibility and connectivity variables (including the presence of a metro station, number of bus stops, and average street length) have a statistically positive effect on pedestrian activity. Only one variable, the percentage of major arterials, had a negative effect.

As explained above, there has been diverse research on this subject that has used PVS data from Seoul between 2009 and 2015, owing to the strength of the research data. The data collected from different years have been used, and the subjects have also varied. By using 2009 PVS data, Kang [17] analyzed the effects of spatial accessibility and centrality on weekday and weekend pedestrian volume, and Sung et al. [56] focused on the effect of the zoning type. Sung et al. [16] tested Jacobs’s theory [13] on street life by using 2010 PVS data. Lee et al. [57] and Jang et al. [20] classified the type of street-based on the surrounding land use and analyzed the effect of the built environment on pedestrian volume by street type by using 2009 and 2012 PVS data, respectively. Lee and Koo [58], Lee et al. [59], and Lee et al. [60] focused on geographical differences. Focusing on the surrounding areas of eleven major subway stations in Seoul, Lee and Koo [58] examined the determinants of pedestrian volume by day of the week and time of the day. Lee et al. [60] studied the differences between arterial roads and narrow streets without a sidewalk, focusing on the three main business districts in Seoul. Lee et al. [59] compared the effects of the physical environment on pedestrian volume by geographically subdividing Seoul into five sub-regions.

As explained so far, the interests of previous researchers have been very distinctive. However, the key findings on the effect of the built environment are generally coincidental. First, density-related variables have a positive association with pedestrian volume. Second, transit accessibility is also a key explanatory factor of pedestrian volume. While most papers applied this variable in the form of the existence or several transit stops (stations) within certain areas or distance to the nearest stops (stations), Jang et al. [20] additionally considered the number of subway station entrances, bus lines, and daily services. Meanwhile, Rodríguez et al. [54] and Lee et al. [59] used transit ridership, which is a more straightforward variable, instead of the accessibility variables. Third, commercial land use and mixed-use are positively associated with pedestrian volume. Forth, higher street connectivity [55] and centrality [17] are also related to more pedestrian volume. Lastly, for the detailed street conditions, wider sidewalks and the existence of nearby crossing encourage more walking; conversely, slopes discourage it. Thus, these significant factors are considered in our regression models. 

#### 2.2.2. Weather and Atmosphere Conditions and Pedestrian Volume 

While several studies have examined the relationship between the physical environment and pedestrian volume, the impacts of weather and atmospheric conditions have been rarely considered. Some studies have explored the impacts of weather conditions on individual-level walking behaviors as well as street- or city-level pedestrian volume. They revealed that higher temperatures encourage more walking, while higher humidity and precipitation in general discourages walking [61,62,63]. In contrast, Shaaban and Muley [64] demonstrated that a higher temperature rather decreases pedestrian volume in a hot climate condition such as Doha, Qatar. In addition, a quadratic relationship can be found between temperature and pedestrian volume; that is, very cold and hot temperatures reduce walking [62]. Accordingly, the impacts of temperature can vary depending on the climate condition of the study area and season.

Depending on the type (purpose) of walking, the weather impacts can also vary. Cools et al. [65] demonstrated that poor weather could lead to cancellations of shopping and leisure trips (discretionary walking trips), and postponement (time-of-the-day change) and route changes of work/school-related trips (mandatory walking trips). Vanky et al. [66] also revealed that weather conditions are more strongly associated with weekend and discretionary travel than with weekday and mandatory travel. However, they are less related to the duration of the walking trip after the trip was initiated [66].

Although many studies have investigated sustainable urban forms that minimize air pollution [67,68,69,70], little is known about the relationship between air quality and outdoor activities, and most of the previous research has focused only on the health impacts of air quality [8,9]. As walking is the most vulnerable travel mode for exposure to air pollution [71] particularly in compact urban contexts such as Seoul, Korea [11,72,73], we can expect that severe air quality directly reduces pedestrian volume. Some preference studies have determined that people are more likely to cancel or postpone their outdoor schedule in the case of bad air quality [74,75]. However, few studies have empirically verified this tendency with revealed preference data (i.e., real air pollution and behavior data). Using measured PM level data in Seoul, Yoon [76] demonstrated that higher PM_10_ decreased sales revenue after the PM_10_ level becomes worse than the “Bad” level. Although sales revenue is highly associated with pedestrian volume, they are not exactly the same, and thus, for this subject, more empirical research is required. 

### 2.3. Limitations of Previous Studies and Research Questions

In short, although various approaches have been adopted to reveal the key determinants of pedestrian volume in urban streets, there have been shortcomings in terms of both the methods and research subjects. For the methods, although most surveys were not conducted on a single day, previous studies have not controlled for time-dependent variables, such as the weather and atmospheric conditions of the survey day. In addition, only a few studies controlled for the potential floating population of the surrounding areas, such as transit ridership. These two factors, therefore, were controlled for in our analysis. 

For the research subjects and content, while the impacts of physical environments have been thoroughly investigated, little is empirically known about the impact of air quality (such as PM_10_) on the pedestrian volume on urban streets. Few studies have explored the micro-scale spatial variations of air pollution and pedestrian volume.

Against this backdrop, this study aims to address the following three research questions. First, we investigate how empirically whether air quality (focusing on PM concentration) affects pedestrian volume in microscopic urban spaces (focusing on a street). If it is influential, our second and third research questions would be about why and how it affects pedestrian volume. These two questions are rooted in the hypothesis that people may react to air quality forecasting or alerts provided by the government and various types of media, as people enact self-protective and information-seeking behaviors to avoid poor air conditions [75]. Accordingly, as a second research question, we analyze whether spatial units of measurement, aggregation, and alert can affect the size of the PM_10_ impact. Finally, as the third research question, we examine whether people’s behaviors vary depending on the content of the information provided (i.e., alert/warning grades), as the third research question. The specific research questions are described in Section 3.2.2, along with the model specifications.

## 3. Empirical Setting

### 3.1. Study Area

The study area covers the capital of South Korea, Seoul, which consists of 25 autonomous boroughs (Gu). The urban form of Seoul is relatively sustainable [17,77], but it is a densely populated area, where 19.4% of the Korean population (9.9 million people) occupy only 0.6% of the national territory (605.3 km^2^) in 2015 [78]. Its gross density is about 17,013 persons/km^2^, approximately 1.5 times that of New York and 3.1 times that of London [17]. Due to its compact, mixed-use, and transit-oriented urban context, the modal share of public transit, walking, and cycling is relatively high at 72.8% in 2016 [79]. As a result, Seoul is one of the most sustainable cities in the world [17].

Nonetheless, Seoul suffers from serious air pollution problems. In 2015, 102 days exceeded the WHO standard of PM_10_ (≤ 50 µg m^−3^), which is nearly one-third of the year [80]. The nation-wide yearly average of PM_2.5_ concentration is also quite high, and this was 29 µgm^−3^ in 2015, which was the second highest among the OECD countries after Turkey and twice the average (15 µg m^−3^). In fact, air pollution is one of the most serious issues for both the government and citizens in Seoul; therefore, it is a very suitable area for this study.

### 3.2. Data, Variables, and Model Specification 

To analyze the relationship between PM_10_ level and pedestrian volume on the streets, a series of multiple regression analyses were applied, using two main sets of public data in Seoul: (1) air quality and meteorological data and (2) Pedestrian Volume Survey (PVS) data. The following subsections describe the model specification of the regression analyses, the measurement methods, and the definition of the variables.

#### 3.2.1. Pedestrian Volume (Dependent Variable)

To measure pedestrian volume (the dependent variable) on the streets, this study uses 2015 PVS data in Seoul. The Seoul city government (Seoul Data Center) and National Information Society Agency in Korea jointly launched this survey in 2009 to investigate street-level pedestrian volume for better data-driven policies and decision-making, and it was annually conducted until 2015. Namely, the data we used were the most recent and last investigated.

In the 2015 survey, 1223 spots (streets) were selected through a preliminary field survey. They covered a considerable portion of Seoul and were relatively evenly distributed. Each street was investigated three times a week (Tuesday or Thursday, Friday, and Saturday) from October 2 to October 31. From 07:30 to 19:30, every pedestrian was counted, and this was aggregated every five minutes.

The data cleaning was as follows. First, the data gathered from the Thursday survey was excluded because it was a supplementary survey for the Tuesday survey. It was conducted on only two days (October 15 and 22), and the number of spots included was quite small (one and three spots, respectively). Second, we also excluded the data surveyed from October 28 to 31, which was not provided by the Seoul Open Data Plaza [81] due to data processing errors. Finally, as a dependent variable, we used the daily pedestrian volume from 2990 observations surveyed in 1207 spots. To convert the distribution closer to normality, a natural log-transformation was applied.

The 2015 PVS data also contains exact X- and Y-coordinates and various physical attributes of the survey spots, allowing us to analyze the relationship between the physical environment and pedestrian behavior. However, as mentioned above, it was not surveyed on a single day; thus, we needed to consider other external factors that might vary depending on the survey date.

#### 3.2.2. Weather and Atmospheric Condition (Test Variables) and the Model Specifications

To measure weather and atmosphere conditions (key test variable) of each street on the PVS days, this study used air quality and meteorological data in Seoul, October 2015. Widely used data-producing methods for these data generally fall into four mains groups: (1) measured data, (2) spatial interpolation, (3) regression modeling, and (4) atmospheric dispersion modeling [76]. In Korean public policy arena, two approaches are mainly used. For short-term forecasting, the Korean Environment Corporation (KEC) and Korea Meteorological Administration (KMA) use predicted values from various atmospheric dispersion modeling and numerical weather prediction modeling systems including the Korean Air Quality Forecasting System [76]. Both systems are used to predict air quality and weather conditions of the following two consecutive days [82]. The prediction is officially announced four times each day (05:00, 11:00, 17:00, and 23:00) through various channels including privately operated mobile applications and mass media, as well as official public websites such as the Air Korea [83], KMA [84], and Seoul Metropolitan government websites [85]. The spatial resolution of the short-term predictions is approximately 1.5–3 km. The forecasting is made using values aggregated at the city or Gu (autonomous boroughs) level for air quality and dong (the smallest administrative unit) level for weather conditions [76,82].

**Figure 1:**
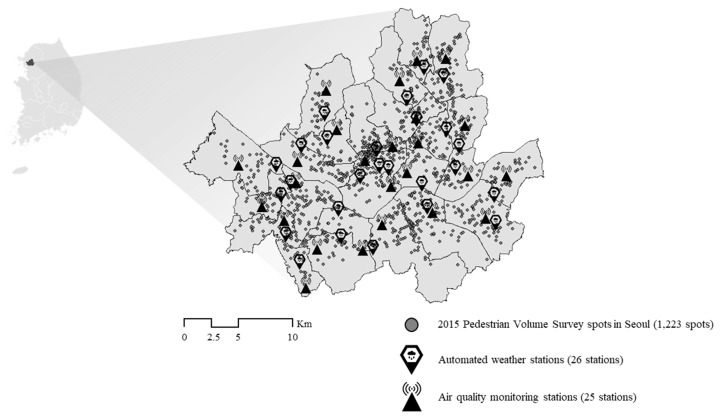
Air quality monitoring stations and pedestrian volume survey spots in Seoul.

This short-term forecasting may help people plan their schedules, affecting their intention to go outside and walk. However, their final decision relies on real-time conditions, and people can obtain the atmosphere conditions of where they are located easily and precisely in real-time using GPS and mobile phones. Therefore, public organizations use measured data for (quasi) real-time alerts and warnings. This study has also mainly used measured data; spatially interpolated data was also partially used, but it is also based on the measured data. Measured air quality data covering PM_10_, PM_2.5_, SO_2_, NO_2_, CO, and O_3_ were gathered from air quality monitoring stations (AQMSs) run by KEC in real-time, and their hourly average levels are publicized every hour on the hour through the diverse channels mentioned above. The KMA and most local governments operate automated weather stations (AWSs) that log temperature, precipitation, humidity, wind direction, and speed data at the near-ground level and these are then provided to the public in ten-minute cycles. In Seoul, 39 AQMSs are currently operating. Among them, as shown in Figure 1, we only used the data from 25 urban AQMSs, one station in each of the 25 autonomous boroughs (Gu), because the other 14 stations specifically aim to measure roadside air quality. Next, among the 56 AWSs in Seoul, we only used the data from 25 AWSs managed by the Seoul metropolitan government that represent each Gu in Seoul and one reference AWS located in Namsan mountain. The other 30 AWSs managed by the KMA were excluded.

Among the various measures explained above, the daily average PM_10_ concentration, daily lowest air temperature, and daily total precipitation of each street on the PVS days were selected as the key test variables. Although PM_2.5_ has received a great deal of attention recently, we only focused on the PM_10_ due to the low accuracy of measuring PM_2.5_ from the official AQMSs in Seoul. While PM_10_ data have been collected since 1995, PM_2.5_ data were not measured until 2015 and still have many missing values because of the low accuracy of sensors. Although PM_2.5_ is not considered, this study can draw implications for it because PM_2.5_ and PM_10_ are almost proportional to each other in Seoul (authors’ calculation using the data from AQMSs).

In addition to PM_10_, the lowest air temperature and precipitation were selected for analysis. Aultman-Hall et al. [62] demonstrated that cold temperatures and rainfall directly and continuously reduce the aggregate level of walking. Although the other temperature data (average and highest) measured in AWSs also affect people’s walking behavior, only the lowest temperature variable was applied to the model to avoid multicollinearity problems. As the average temperature in October 2015 in Seoul, 17.24 °C, was quite low, we could predict that people are more sensitive to the minimum temperature than the maximum temperature as demonstrated by Shaaban and Muley [64] and Vanky et al. [66]. The variables used were also log-transformed to convert their distribution closer to normality.

To address the research questions of this study explained in Section 2.3, the regression models were diversified based on the weather and atmospheric condition variables, particularly PM_10_. As explained above, the questions generally stemmed from the assumption that people behave based on the information they receive from the government and the media.

First, the effects of weather and atmospheric conditions on pedestrian volume can vary depending on how they are measured and how people are notified. In this way, the spatial unit of measurement, aggregation, and alerts can be a dominant factor because people may choose their behaviors based on the official forecast or real-time information provided by the government media rather than their senses. Thus, this study applies three different regression models depending on the spatial measurement unit of weather and atmospheric condition variables: Si (a city in Korean), Gu (a borough in Korean), and a 30 m × 30 m cell. Weather and atmospheric conditions were originally measured from the AWS and AQMS of each Gu, and therefore, they were directly used as a representative value of each Gu. Si-level variables were also defined using the information that was officially measured and announced by government organizations. Although more micro-level information was not provided, it may affect people’s behaviors. Therefore, we applied additional cell-level (30 meters × 30 meters) weather and atmospheric condition variables that were estimated using the Kriging ArcGIS technique based on the values of the 26 AWSs and 25 AQMSs. Among the three levels of variables, only Si and Gu-level information can be accessed in various ways, cell-level information cannot be obtained, and the differences in the values of each cell cannot be precisely predicted or recognized [74]. Thus, we can expect that people, and hence the pedestrian volume, may respond sensitively to the Gu-level information.

Second, people’s behaviors can vary depending on the content of the information provided (i.e., alert/warning grades based on air pollution intensity). Hence, we applied three stratified regression models based on the grade of the PM_10_. PM_10_ concentrations are presented as a numerical value, but they are also provided as grades with warning images so that people can easily recognize them. The Ministry of Environment in Korea uses a four-grade alert/warning system for PM_10_ based on the expected health concerns: “Good” (below 30 µg m^−3^), “Normal” (30–80 µg m^−3^), “Bad” (80–150 µg m^−3^), and “Very Bad” (over 150 µg m^−3^). We reclassified these as “Good,” “Normal,” and “Bad” (over 80 µg m^−3^) because the value did not exceed 150 µg m^−3^ on the PVS days. We can expect that people react more sensitively to PM_10_ level when the grade is worse [76].

All the regression models described above applied as a form spatial regression (spatial-lag or spatial-error models of GeoDaSpace software, The University of Illinois at Urbana-Champaign, Illinois, The United States of America) to consider spatial autocorrelation of pedestrian volume data. 

#### 3.2.3. Physical Environment and Other Control Variables

To examine the impacts of the key test variables described above, we controlled for other dominant factors that could explain pedestrian volume on the streets. As illustrated in Table 1, we classified the type of walking that determines pedestrian volume based on the outdoor activity typology [86] and whether pedestrian’s current location is the origin, destination, or route of the passage. Table 1 also illustrates some of the determinants of pedestrian volume (i.e., control variables) on the streets and their expected explanatory power by the type of walking. 

Most of all, the fixed and floating population of the surrounding areas of survey spots were expected to explain the variation in pedestrian volume more than the other variables, as they can be directly converted into pedestrians on the streets. In addition, as they can be measured in various ways, we considered as many approaches as possible with the data available. First, we controlled for the number of residents and workers in the surrounding areas (i.e., fixed population) and this was expected to explain the variations in walking trips and other discretionary activities where the origin or destination was the survey site or its surrounding area (type A, B, and D; see Table 1). To this end, the population and job density of the census tract, where the survey site is located, were applied in the models, and because the area of the census tract varies, we used density-type variables instead of the total number of residents and workers.

Second, we also controlled for floating populations, including wandering and passing-by pedestrians moving to specific destinations or transit stations. By controlling for the transit ridership, we could additionally explain the variations in wandering and other related activities where the destination or route is in the survey site or its surrounding area (type C and E) as well as type A and B explained above (see Table 1). Using Smart Card Data in Korea, the total number of passengers boarding and getting off at all bus stops and subway stations within 400 meters (quarter-mile) of the survey spot on survey day was measured as a control variable in the model. As public transit users include a significant proportion of residents or workers in the area, the first and second approaches are not entirely exclusive. However, because the PVS was conducted over several days, it is appropriate to reflect the number of transit users on each survey day as a way to explain the pedestrian volume variables that vary from day-to-day. Along with weather and atmospheric condition variables, this can partially address the fundamental limitations of prior studies that have used the PVS data. The four log-transformed population-related variables (population and job density and bus and subway ridership) can be expected to explain the variations in pedestrian volume extensively.

Next, as argued by Gehl [86], the quality of the physical environment can also affect the number of people on a street. To control for land use of the surrounding area, three dummy variables volume (i.e., control variables) on the streets were used, with green areas as the reference group. In Korea, all land in urbanized areas is designated as one of these four land-use types, and the uses cannot overlap. In general, people tend to walk and stay more in commercial streets with a variety of tenants [20,59].

As control variables, we also applied street type and the specific physical conditions of the survey spots. As this information is included in the PVS data, most previous papers also controlled for them. With regard to street type, “streets with sidewalk” and “streets without a sidewalk (shared street)” were defined as dummy variables, and “streets with a sidewalk, but shared with bicycles” functioned as a reference group in the model. When it comes to street condition, we controlled for sidewalk width, number of traffic lanes, and a series of dummy variables such as the presence of a centerline, obstacles, braille blocks, slopes, fences, and crosswalks. Regarding the presence of obstacles, the PVS data defines all types of street furniture as obstacles to walking, and previous papers have used it in this way [59,87,88]. We, therefore, reclassified this into street furniture and obstacles. Street furniture includes essential elements for creating a pedestrian friendly environment such as streetlights, road signs, letterboxes, and trees. However, obstacles include undesirable street elements that obstruct the safe and convenient passage of pedestrians on the street, including on-street parking, illegal newsstands, and standing signboards. Here, these dummy variables were measured based on their presence within 50 m from the survey spot, and the presence of slopes was entirely determined by the trained investigator’s perception. These physical environment variables partially or extensively explained the variations in the outdoor activities.

Lastly, the day of the week of the PVS was also considered: Friday and Saturday were dummy variables, and Tuesday was a reference group. Depending on the location, the impact of the day of the week was expected to vary. In general, pedestrian volume of the inner-city area is expected to be larger during the weekdays compared to the weekend.

As explained above, the dependent variable (pedestrian volume) and three main test variables (PM_10_ concentration, lowest air temperature, and precipitation) were log-transformed to convert their distribution closer to normality. In other words, the regression analysis adopted a double-log function; thus, we could interpret the coefficients of the weather and atmospheric condition variables as the elasticity of pedestrian volume.

## 4. Results 

### 4.1. Preliminary Analysis: PM_10_ Concentration and Pedestrian Volume in Seoul

**Figure 2:**
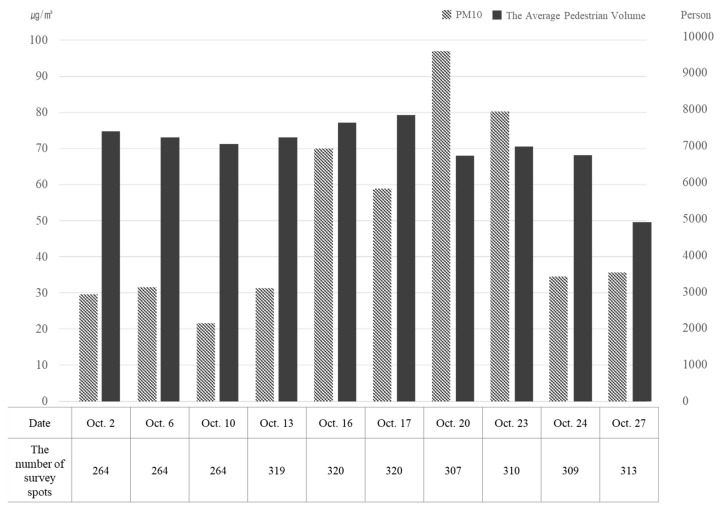
Average particulate matter (PM)_10_ concentration and pedestrian volume by the date of the survey (October 2015; source: Authors’ calculations using air quality and PVS data in Seoul).

Figure 2 illustrates average PM_10_ concentration and pedestrian volume over ten PVS days in October 2015. As shown, whereas daily pedestrian volume was relatively constant except for October 27, PM_10_ concentration was volatile to some extent. It was difficult to ascertain any apparent tendency toward the relationship between both variables.

**Figure 3:**
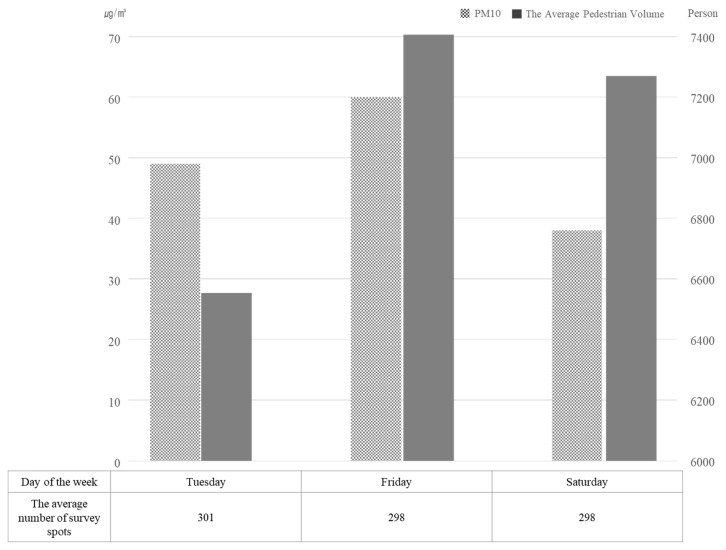
Average PM_10_ concentration and pedestrian volume by the day of the week (October 2015; source: Authors’ calculations using air quality and PVS data in Seoul).

The result of averaging by day is shown in Figure 3. It illustrates that the average daily pedestrian volume on weekdays (Tuesday) was smaller than on weekends (Friday and Saturday). In particular, the pedestrian volume on Friday was the largest, although it had the highest PM_10_ concentration level. 

**Figure 4:**
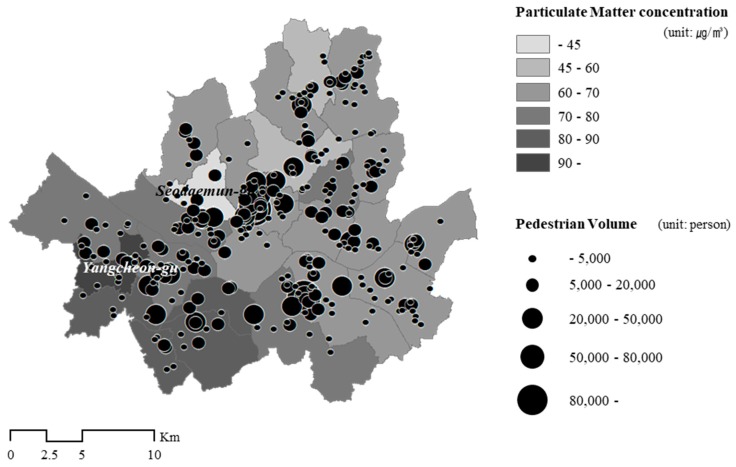
Gu-level PM_10_ concentration and pedestrian volume (October 16 2015; source: Authors’ calculations using air quality and PVS data in Seoul).

To examine the spatial relationships between the two variables in detail, we mapped Gu-level PM_10_ concentration and pedestrian volume on a randomly selected single day, October 16 2015 (Figure 4). Figure 4 illustrates that the PM_10_ concentration level was higher in the southwestern area than in the northeast area of the city. While Seodaemun-gu had the lowest concentration, Yangcheon-gu was ranked the highest. However, the locations of the pedestrian hub (larger than 50,000) seemed to be irrelevant to the PM_10_ hot spots. Rather, they were concentrated in three main business districts in Seoul: Jongro-gu, Gangnam-gu, and Yeongdeungpo-gu. In contrast, the northeast areas with clean air were dominated by residential use and had a small volume of pedestrians. This implies that the pedestrian volume on the streets was also determined by other external factors besides air quality. Therefore, we tested the impact of air quality after taking other factors into account by applying the spatial regression approach.

### 4.2. Impact of Weather and Atmospheric Condition on Pedestrian Volume by the Spatial Unit of Alert

Table 2 and Appendix A respectively present the series of spatial and ordinary least squares (OLS) regression results of the log-transformed daily pedestrian volume by spatial measurement unit of weather and atmospheric condition variables. Moran’s I values, presented in Appendix A, were statistically significant in all models, indicating the presence of spatial autocorrelation. Accordingly, we applied both spatial-lag and spatial-error models, and found that the spatial-error model has a better goodness of fit than the spatial-lag model based on the Lagrange Multiplier (LM) lag and error test. The lambda (λ) values in Table 2 indicated spatial autocorrelation across survey spots for pedestrian volume. The explanatory power (R-square) of the spatial-error models (0.539 in average) was larger than the OLS models (0.417 in average) and those of previous papers [16,17,20,56,59] that used the same data. Although the variance inflation factor (VIF) values are not reported in Table 2 and Appendix A owing to the limited space, no multicollinearity was found.

In the Gu level model, the PM_10_ concentration variable was negatively and significantly associated (at *p*-value < 0.05) with the pedestrian volume on the survey day, after considering the other factors. This implies that PM_10_ concentration can discourage people’s outdoor activities in a microscopic urban space like a street (the first research question), and it provides empirical evidence to add to the existing body of literature on air pollution and outdoor activities. 

To address the second research question, we compared the elasticities of pedestrian volume in each model and their explanatory power. As shown in Table 2, the PM_10_ elasticity of pedestrian volume (walking demand) was larger in the Gu-level model than in the Si- and cell-level models. The Gu-level model demonstrated that when the PM_10_ concentration increased by 1%, the amount of daily pedestrian volume on the streets decreased by 0.121%. In addition, the explanatory power (R-square) of the Gu-level model (0.121) was also slightly larger than the other two models (0.085 and 0.064), although the difference was negligible. We tentatively conclude that people respond more sensitively to the Gu-level information of PM_10_ concentration, as expected.

The results on the other weather variables also demonstrated similar patterns. The Gu-level model demonstrated that when the precipitation increased by 1%, the pedestrian volume decreased by −0.029%, respectively. This reconfirms previous empirical evidence [62,64,66]. The Si- and cell-level models also showed similar results.

These results imply that people are more likely to consider Gu-level weather and air quality information (the smallest spatial unit of real-time air quality alert) when they decide their outdoor activities. This is simply because a large number of people’s daily activities are confined to specific regions, and not the whole city of Seoul, and the atmospheric conditions of Seoul are different by region, as shown in Figure 4. Based on the results, we also suggest that people cannot accurately recognize the atmospheric conditions of microscopic units of space (smaller than Gu), as demonstrated by Semenza et al. [74], or even if they do, the effect of the information on their behavioral choices is limited. So, in reality, it is impossible for ordinary people to recognize or predict real-time differences between the atmospheric conditions of different regions, and subsequently, people’s behavior is highly dependent on the information that is publicized. Oltra and Sala [75] (p.869) argued that “attention to air quality levels and worry are important predictors of self-protective and information-seeking behavior”. Therefore, the spatial unit of forecast or real-time alert is expected to have a large role in people’s reaction to PM_10_.

The results of the other control variables were generally as expected. Focusing on the Gu-level model, the key results were interpreted as follows. First, different types of population and land-use variables were significantly associated with pedestrian volume at the significance level of 0.01. In particular, the public transit ridership variables, which represent the floating population of the surrounding areas, constituted the independent variables that best explained the dependent variable. The results demonstrated that the pedestrian volume on the street increased by 0.266 and 0.040 percent, respectively, when the number of bus and subway users within 400 meters from the survey spot increased by 1 percent. Most of the previous studies have revealed that higher accessibility induces a larger volume of pedestrians on the streets. However, this study more directly reflects the daily floating population using transit ridership variables, while previous studies has applied its driving forces (i.e., physical facilities) in the form of the existence or density of transit stops, or distance to the nearest stops [17,56,57,59]. The existing approaches may be better to draw policy implications for public transportation facilities (such as how close and how much should be deployed). However, our approach, which can more accurately explain the variance in the daily amount of walking, would be desirable, if we aimed to better control for the determinants of pedestrian volume in order to more precisely grasp the impacts of the key variable (i.e., PM_10_). In other words, our approach is better for understanding the impacts of PM_10_ on pedestrian volume. 

Among two fixed population variables, which were defined as the population and job density of the census tract, only population density showed a negative association with the pedestrian volume. It simply indicates areas with high population density would represent outlying residential areas, and, of course, less people walk in those areas than in inner-city areas.

The land-use variables also demonstrated reliable results. As previous studies revealed [17,20,56,59], commercial, residential, and industrial land attracts a larger amount of people, compared to green areas. For the street type variables, the results suggest that “streets with pedestrian-only sidewalk” and “streets without a sidewalk (i.e., shared street)” are more likely to attract more pedestrians, compared to “streets with pedestrian/bicycle mixed sidewalks”. This implies that Korea’s policy of sharing pedestrian space with bicycles instead of providing exclusive lanes for bicycles has led to a more dangerous environment than mixed spaces with pedestrians and vehicles. However, no empirical consensus has been reached on this subject in previous studies because they used PVS data from different years that defined the type of street differently.

Lastly, the street condition (i.e., street design quality) variables also affected the pedestrian volume. As expected, and as previous papers have consistently revealed, wider streets provide more favorable environments for pedestrians, whereas sloping streets do not. In addition, the results demonstrated that streets with nearby crosswalks and a larger number of traffic lanes accommodate more pedestrians, and this is consistent with all of the previous studies that have used PVS data. Crosswalks can attract and guide people, and a larger number of traffic lanes represent a higher hierarchy of the street. The presence of fences is also positively associated with a larger volume of pedestrians. This means that when the other conditions are the same, more people want to walk in a safer environment where independent pedestrian space is secured. This has been verified by Jang et al. [20].

In contrast, the results revealed that the presence of centerlines is negatively associated with pedestrian volume. This is related to specific road conditions in Seoul. Seoul has many narrow streets without a sidewalk, which are called i-myeon-do-ro in Korean (back roads), and pedestrians and vehicles share these streets [89]. In this context, people tend to perceive the i-myeon-do-ro without centerlines as more pedestrian-oriented spaces, because centerlines are a typical symbol of streets for vehicles [90,91]. In contrast, when the other dimensions are the same, people may regard i-myeon-do-ro with centerlines as streets for motorized transportation, and this could limit safe and convenient walking [90,91]. Next, whereas the coefficient of the street furniture variable, which was expected to encourage street activities, was insignificant, the presence of obstacles, which was expected to disturb continuous walking, was positively and significantly associated with pedestrian volume. These seem to be contradictory to our expectation and general knowledge. However, Lee et al. [60] also demonstrated that pedestrian volume could be large in places with specific obstacles. This means that the street facilities that we considered to be obstacles might be a physical feature of pedestrian-concentrated areas.

### 4.3. Comparing the Coefficients of PM_10_ in Three Stratified Models by Grade

To address the last research question of whether PM_10_ alert/warning grade affects the level of PM_10_ elasticity of pedestrian volume (walking demand), three stratified models by grade were applied. The models included the Gu-level weather and atmospheric condition variables and other control variables. The “log precipitation” and “day of the week” variables were excluded because all of the cases equaled zero in the models. This was indispensable as this section aims to compare the coefficients of the three models. 

Consistent with the models in Table 2, we employed OLS regressions (presented in Appendix B) and LM lag and error test. The statistics showed that spatial-lag model has better goodness of fit for “Good” model, while spatial-error model is better for “Normal” and “Bad” models. Table 3 illustrates the results of the three stratified spatial regression models. The adjusted R-squares of all of the models were large enough, and no multicollinearity was found even though some of the variables were excluded. The result of each variable was also similar to the models suggested in Table 2. Thus, only the coefficients of the PM_10_ variables were interpreted.

In “Good” and “Bad” of the models, the PM_10_ concentration variable was negatively and significantly associated with pedestrian volume on the survey day. However, the size of the coefficient (elasticity) varied depending on the models. Specifically, when the PM_10_ level was larger than 80 µg m^−3^ (“Bad” grade), and after taking other factors into account, a 1 percent increase in the average daily PM_10_ was associated with a decrease of pedestrian volume by 1.147%. The decreasing rate (0.652%) was much smaller in the “Good” grade model with a PM_10_ level lower than 30 µg m^−3^. This implies that the worse the PM_10_ level, the more sensitive people are to the increase in PM_10_ concentration, which reduces outdoor activities.

Surprisingly, the result showed that the PM_10_ concentration variable was not significantly associated with pedestrian volume when in the “Normal” (30–80 µg m^−3^). Yoon [76] revealed that as PM_10_ concentration increases, sales revenue increases when the PM_10_ level is lower than 109 µg m^−3^. Based on this result, Yoon argued that “people are indifferent about PM_10_ concentration until the level gets worse than the “Bad” category” [76] (p. 364). These results lead us to the conclusion that the starting point of the “Bad” grade of the PM_10_ level can be a psychological threshold value, leading to people refraining from going out and walking.

## 5. Conclusions

Using 2015 PVS data and air quality data in Seoul, this study investigated the relationships between PM_10_ concentration and pedestrian volume on the streets, focusing on the differences in the spatial unit of alert and the grade of PM_10_. In particular, compared to previous papers using the same public data set, this study captured the PM_10_ elasticity of pedestrian volume more precisely, by controlling for the daily floating population in the surrounding areas of the survey spot using SmartCard data. The key findings and implications of the three research questions are summarized as follows.

First, this study reconfirms the conventional belief that PM_10_ concentration affects people’s intention to walk, and decreases pedestrian volume, particularly on a street level. Specifically, when the PM_10_ concentration increases by 1%, the daily pedestrian volume on the streets decreases by 0.121%. This implies that poor outdoor air quality not only harms people’s health but also depresses the local economy. Thus, political efforts to vitalize streets, invigorate the commercial sector, and revive the local economy by attracting more people must be made alongside efforts to improve the air quality of the city.

Second, the three regression models adopted different spatial aggregation units of air quality variables and suggest that PM_10_ elasticity of the pedestrian volume is larger in the Gu-level model than in the Si- and cell-level models. This demonstrates that people respond more sensitively to Gu-level information of PM_10_ concentration. This is simply because the Gu is the smallest spatial unit of air quality alert that people can access in Korea and people cannot recognize the atmospheric conditions of a microscopic unit of space such as a street [74]. This means that spatial units of alert can be a critical factor that influences people’s intention to walk. If the spatial unit of alert is larger than what people require, people may not be able to identify the exact atmospheric conditions of the specific places where they walk. Accordingly, they may not be aware that the atmospheric condition is worse than the information they acquired, and may damage their health by going ahead with outdoor activities, or conversely, may fail to recognize that the level is better than the alert, and may not be able to perform their activities because they will avoid going outside. Subsequently, air pollution information should be provided in smaller spatial units. Indeed, people are increasingly demanding a higher spatial resolution of PM_10_ information for their health. However, this requires a denser installation of AQMSs, which would lead to higher operating costs. Thus, future studies should find optimal (i.e., cost-and-benefit balanced) spatial units of alert for better policies.

Finally, from the three stratified regression models by PM_10_ alert grade, we found that people are more likely to refrain from walking on a street when the alert reaches the “Bad” grade; the lowest value of the “Bad” grade (i.e., 80 µg m^−3^) can act as a psychological threshold. The “Bad” grade alert and its warning image can also be a definite and effective signal that alters people’s behaviors. This result supports the effectiveness of the policies that have tightened the PM_10_ standards to alleviate the health effects of PM_10_ that have been highlighted in many diverse studies [8,9,92]. In 2018, to enhance public health, the Korean government strengthened air quality forecasting and warning standards for PM_2.5_ in line with international standards. For PM_10_, however, there is still room for improvement because the standards are much less strict than the WHO standards. The empirical evidence highlighted in this paper supports the idea that this approach (i.e., strengthening air quality warning standard for PM) is an effectual measure for public health. 

This study has several shortcomings. Most of all, the two key variables, PM_10_ concentration and pedestrian volume, have high seasonal fluctuations, and due to climate change, the deviation from year to year has been increasing recently. The causes of PM_10_ and its spread also differ greatly from region to region. However, the results presented in this study were limited to the small space-time range of Seoul, Korea, in October 2015. Although we used a relatively large dataset from a metropolitan area to increase its generalizability, it is still limited due to its particular study area and period. Therefore, our findings need to be compared against further research focusing on diverse cities (including different types of streets) and extended periods (including different seasons, days of the week, and time of the day). Fortunately, as part of its smart city initiative, the Seoul Metropolitan Government recently announced a plan to install 50,000 public IoT sensors that measure air quality and pedestrian volume, which is expected to enable more long-term research. 

Next, the analytical approach of this study could not consider the individual characteristics of pedestrians. Accordingly, this study did not determine whether each pedestrian’s actions were determined by the PM_10_ levels. Depending on the purpose of walking, the effect of PM_10_ can also vary. To address this issue, individual-level surveys are required; however, these are costly and time-consuming. As the level of detail of the survey increases, the target areas and samples we can focus on will decrease, and this tradeoff is a fundamental limitation of this field. 

## Figures and Tables

**Table 1:** Determinants of pedestrian volume on the streets and their expected explanatory power by type of walking.

Type of Outdoor Activity [86]	Possible Type of Walking on a Street	Control Variables and Their Expected Explanatory Power			
		Fixed and Floating Populations in Surrounding Areas			Physical Environment
		# of Residents	# of Workers	# of Public Transit Users	
Necessary activities	A. The street or nearby area is the origin of the walking trip	○	○	△	△
	B. The street or nearby area is the destination of the walking trip	○	○	△	△
	C. The street or nearby area is on the route of the walking trip			△	△
Optional and social activities	D. The street or nearby area is the origin of the wandering and other related activities	○	○		○
	E. The street or nearby area is the destination or on the route of the wandering and other related activities			○	○

○: This variable is expected to explain the variation of pedestrian volume on the streets extensively; △: This variable is expected to explain the variation of pedestrian volume on the streets partially; ## of residents, # of workers: # of people who live or work in nearby areas; ## of public transit users: # of people who take, transfer, or get off the bus or subway in nearby areas (areas of 400 m from the Pedestrian Volume Survey (PVS) center).

**Table 2:** Spatial regression models of log-transformed daily pedestrian volume by the spatial alert unit of weather and atmospheric condition variables.

Variable	Si-Level Model		Gu-Level Model		Cell-Level Model	
	Spatial Error		Spatial Error		Spatial Error	
	Coef.	z	Coef.	z	Coef.	z
Lambda (λ)	0.707 ***	29.167	0.706 ***	29.070	0.708 ***	29.262
Constant	6.755 ***	5.877	6.476 ***	5.526	6.766 ***	5.840
Weather and atmosphere condition						
log_PM_10_ concentration	−0.085	−1.631	−0.121 **	−2.286	−0.064	−1.232
log_lowest temperature	0.022	0.223	0.152	1.233	−0.023	−0.201
log_precipitation	−0.035 **	−2.083	−0.029 *	−1.824	−0.033 **	−2.028
Population						
log_population density	−0.211 **	−2.072	−0.206 **	−2.022	−0.209 **	−2.052
logjob density	−0.029	−0.524	−0.025	−0.447	−0.029	−0.821
logbus ridership	0.266 ***	12.538	0.266 ***	12.536	0.266 ***	12.511
logsubway ridership	0.041 ***	10.890	0.040 ***	10.880	0.040 ***	10.882
Land use						
Residential	0.342 ***	3.665	0.341 ***	3.645	0.3417 ***	3.657
Commercial	0.481 ***	4.656	0.481 ***	4.653	0.481 ***	4.650
Industrial	0.238 *	1.768	0.241 *	1.787	0.238 **	1.762
Street type						
With a sidewalk	0.616 ***	10.764	0.615 ***	10.754	0.615 ***	10.744
Without a sidewalk (shared with pedestrians and vehicles)	0.530 ***	7.088	0.531 ***	7.096	0.529 ***	7.070
Street condition						
Sidewalk width	0.062 ***	7.846	0.062 ***	7.837	0.062 ***	7.831
# of traffic lanes	0.020 **	2.389	0.021 **	2.424	0.020 **	2.372
Presence of centerline	−0.119 **	−2.045	−0.120 **	−2.059	−0.118 **	−2.020
Presence of street furniture	−0.078	−1.356	−0.077	−1.348	−0.078	−1.348
Presence of obstacle	0.378 ***	5.734	0.377 ***	5.720	0.379 ***	5.750
Presence of braille block	0.029	0.797	0.027	0.773	0.030	0.826
Presence of street slope	−0.289 ***	−7.214	−0.290 ***	−7.232	−0.290 ***	−7.229
Presence of fence	0.139 ***	3.576	0.138 ***	3.562	0.139 ***	3.578
Presence of crosswalk	0.207 ***	5.426	0.207 ***	5.420	0.207 ***	5.425
Day of the week						
Friday	0.011	0.299	0.022	0.601	0.017	0.449
Saturday	0.055	1.329	0.037	0.892	0.068*	1.650
Summary Statistics						
N	2990					
Adjusted R-square	0.539		0.539		0.539	
Robust LM error	324.154 ***		314.508 ***		328.838 ***	

Note: The reference group of Land use, Street type and Day of the week are “Green,” “With sidewalk, but shared with bicycles” and “Tuesday,” respectively. * Significant at *p* < 0.1; ** Significant at *p* < 0.05; *** Significant at *p* < 0.01.

**Table 3:** Spatial regression models of log-transformed daily pedestrian volume by the grade of PM_10_.

Variable	Good (< 30 µg m^−3^)		Normal (30 µg m^−3^ ≤ PM_10_ < 80 µg m^−3^)		Bad (≥ 80 µg m^−3^)	
	Spatial Lag		Spatial Error		Spatial Error	
	Coef.	z	Coef.	z	Coef.	z
Rho (ρ)	0.411 ***	5.867				
Lambda (λ)			0.559 ***	16.777	0.516 ***	8.218
Constant	2.904	1.562	5.420 ***	4.384	2.619	0.533
Gu-level weather and atmosphere condition						
log_PM_10_ concentration	−0.652 ***	−2.610	−0.072	−1.005	−1.147 **	−2.117
log_lowest temperature	0.708 ***	3.051	0.106	0.834	1.132	0.936
Population						
log_population density	−0.066	−0.475	−0.239 **	−2.333	0.276	1.511
log_job density	−0.067	−1.156	0.075	1.291	−0.045	−0.436
log_bus ridership	0.251 ***	6.652	0.318 ***	11.829	0.302 ***	6.448
log_subway ridership	0.045 ***	5.798	0.039 ***	8.477	0.052 ***	6.726
Land use						
Residential	−0.070	−0.309	0.325 ***	2.836	0.455 **	2.031
Commercial	0.100	0.407	0.518 ***	4.108	1.010 ***	3.854
Industrial	0.033	0.117	0.244	1.474	0.386	1.352
Street type						
With sidewalk	0.712 ***	5.463	0.610 ***	8.385	0.915 ***	6.849
Without sidewalk (shared with pedestrians and vehicles)	0.425 **	2.125	0.516 ***	5.562	0.804 ***	4.779
Street condition						
Sidewalk width	0.064 ***	3.025	0.062 ***	6.499	0.109 ***	5.987
# of traffic lanes	0.057 ***	2.758	0.011	1.019	0.031 *	1.697
Presence of centerline	−0.241 *	−1.668	−0.202 ***	−2.759	−0.123	−0.940
Presence of street furniture	−0.267*	−1.868	−0.036	−0.491	0.152	1.114
Presence of obstacle	0.301*	1.894	0.438 ***	5.249	0.641 ***	4.152
Presence of braille block	−0.111	−1.355	0.095 **	2.096	−0.011	−0.125
Presence of street slope	−0.180 **	−2.060	−0.326 ***	−6.407	−0.129	−1.307
Presence of fence	0.315 ***	3.340	0.099 **	2.014	0.078	0.802
Presence of crosswalk	0.298 ***	3.221	0.147 ***	3.093	0.255 **	2.811
Summary Statistics						
N	608		1874		508	
Adjusted R-square	0.456		0.559		0.550	
Robust LM lag/error	14.6429 ***		74.0529 ***		21.4013 ***	

Note: The reference group of Land use, Street type and Day of the week are “Green,” “With sidewalk, but shared with bicycles” and “Tuesday,” respectively. * Significant at *p* < 0.1; ** Significant at *p* < 0.05; *** Significant at *p* < 0.01.

## Appendix A. Multiple Regression Models of Log-Transformed Daily Pedestrian Volume by the Spatial Alert Unit of Weather and Atmospheric Condition Variables

**Variable**	**Si-Level Model**		**Gu-Level Model**		**Cell-Level Model**	
	**OLS**		**OLS**		**OLS**	
	**Coef.**	**t**	**Coef.**	**t**	**Coef.**	**t**
Constant	5.062 ***	7.937	4.314 ***	6.521	4.839 ***	7.499
Weather and atmosphere condition						
log_ PM_10_ concentration	−0.169 ***	−2.912	−0.247 ***	−4.339	−0.149 ***	−2.609
log_lowest temperature	0.076	0.713	0.419	3.217	0.102	0.803
log_precipitation	−0.059 ***	−3.151	−0.051 ***	−2.872	−0.058 ***	−3.188
Population						
log_population density	−0.194 ***	−3.734	−0.178 ***	−3.423	−0.186 ***	−3.592
log_job density	0.076 ***	2.753	0.081	2.922	0.078	2.821
log_bus ridership	0.330 ***	17.500	0.329 ***	17.440	0.330 ***	17.436
log_subway ridership	0.047 ***	13.558	0.047 ***	13.566	0.047 ***	13.540
Land use						
Residential	0.309 ***	3.214	0.304 ***	3.169	0.307 ***	3.192
Commercial	0.538 ***	5.070	0.542 ***	5.113	0.536 ***	5.046
Industrial	0.319 ***	2.636	0.318 ***	2.627	0.321 ***	2.646
Street type						
With a sidewalk	0.685 ***	11.468	0.683 ***	11.452	0.683 ***	11.427
Without a sidewalk (shared with pedestrians and vehicles)	0.579 ***	7.449	0.579 ***	7.456	0.574 ***	7.377
Street condition						
Sidewalk width	0.083 ***	9.929	0.082 ***	9.935	0.083 ***	9.913
# of traffic lanes	0.017 *	1.941	0.018 **	2.038	0.017 *	1.938
Presence of centerline	−0.223 ***	−3.660	−0.228 ***	−3.749	−0.223 ***	−3.656
Presence of street furniture	−0.026	−0.417	−0.026	−0.419	−0.025	−0.395
Presence of obstacle	0.487 ***	6.975	0.482 ***	6.906	0.487 ***	6.976
Presence of braille block	0.004	0.107	0.004	0.111	0.003	0.099
Presence of street slope	−0.239 ***	−5.740	−0.240 ***	−5.783	−0.240 ***	−5.779
Presence of fence	0.132 ***	3.162	0.132 ***	3.155	0.133 ***	3.168
Presence of crosswalk	0.176 ***	4.371	0.179 ***	4.437	0.178 ***	4.409
Day of the week						
Friday	0.007	0.163	0.019	0.449	0.004	0.098
Saturday	0.076	1.586	0.030	0.646	0.084	1.786
Summary						
N	2,990					
Adjusted R-square	0.417		0.418		0.416	
Log likelihood	−3946.19		−3943.49		−3948.17	
Akaike info criterion	7940.39		7934.98		7944.34	
Schwarz criterion	8084.46		8079.05		8088.41	
Statistics						
Moran’s I	35.616 ***		35.362 ***		35.737 ***	
Note: The reference group of Land use, Street type and Day of the week are “Green,” “With sidewalk, but shared with bicycles” and “Tuesday,” respectively. * Significant at *p* < 0.1; ** Significant at *p* < 0.05; *** Significant at *p* < 0.01.						

## Appendix B. Multiple Regression Models of Log-Transformed Daily Pedestrian Volume by the Grade of PM_10_

**Variable**	**Good (<30 µg m^−3^)**		**Normal (30 µg m^−3^ ≤ PM_10_ < 80 µg m^−3^)**		**Bad (≥80 µg m^−3^)**	
	**OLS**		**OLS**		**OLS**	
	**Coef.**	**t**	**Coef.**	**t**	**Coef.**	**t**
Constant	6.377 ***	3.436	4.530 ***	5.4852	−0.830	−0.193
Gu-level weather and atmosphere condition						
log_ PM_10_ concentration	−0.691 ***	−2.647	−0.176 **	−2.318	−1.213 **	−2.224
log_lowest temperature	0.816 ***	3.421	0.259 *	1.922	2.365 **	2.203
Population						
log_population density	−0.167	−1.161	−0.242 ***	−3.882	0.274 **	1.969
log_job density	−0.017	−0.283	0.127 ***	3.684	−0.010	−0.129
log_bus ridership	0.269 ***	6.875	0.358 ***	14.837	0.303 ***	6.403
log_subway ridership	0.045 ***	5.495	0.045 ***	10.300	0.053 ***	6.486
Land use						
Residential	0.008	0.032	0.350 ***	2.966	0.492 **	2.108
Commercial	0.242	0.941	0.556 ***	4.294	1.070 ***	3.978
Industrial	0.280	0.947	0.307 **	2.028	0.332	1.202
Street type						
With sidewalk	0.702 ***	5.145	0.599 ***	7.890	0.936 ***	6.833
Without sidewalk (shared with pedestrians and vehicles)	0.340	1.627	0.525 ***	5.492	0.857 ***	4.901
Street condition						
Sidewalk width	0.068 ***	3.053	0.074 ***	7.368	0.118 ***	5.840
# of traffic lanes	0.057 ***	2.641	0.012	1.113	0.024	1.215
Presence of centerline	−0.250 *	−1.658	−0.273 ***	−3.605	−0.233 *	−1.672
Presence of street furniture	−0.278 *	−1.856	−0.017	−0.221	0.202	1.393
Presence of obstacle	0.350 **	2.105	0.454 ***	5.225	0.730 ***	4.488
Presence of braille block	−0.125	−1.465	0.057	1.212	−0.001	−0.006
Presence of street slope	−0.173 *	−1.887	−0.287 ***	−5.421	−0.106	−1.060
Presence of fence	0.316 ***	3.206	0.115 **	2.215	0.027	0.262
Presence of crosswalk	0.345 ***	3.576	0.107 **	2.142	0.241 **	2.481
Summary						
N	608		1,874		508	
Adjusted R-square	0.425		0.423		0.488	
Log likelihood	−805.982		−2443.86		−648.26	
Akaike info criterion	1653.96		4929.72		1338.52	
Schwarz criterion	1746.58		5045.97		1427.36	
Statistics						
Moran’s I	9.674 ***		19.758 ***		8.275 ***	
Note: The reference group of Land use, Street type and Day of the week are “Green,” “With sidewalk, but shared with bicycles” and “Tuesday,” respectively. * Significant at *p* < 0.1; ** Significant at *p* < 0.05; *** Significant at *p* < 0.01.						
